# A Review on Viruses Infecting Taro (*Colocasia esculenta* (L.) Schott)

**DOI:** 10.3390/pathogens8020056

**Published:** 2019-04-25

**Authors:** Mohd Shakir Mohamad Yusop, Mohd Faiz Mat Saad, Noraini Talip, Syarul Nataqain Baharum, Hamidun Bunawan

**Affiliations:** 1 Institute of Systems Biology, Universiti Kebangsaan Malaysia, 43600 Bangi Selangor, Malaysia; shakir.yusop@gmail.com (M.S.M.Y.); faizsaad@ukm.edu.my (M.F.M.S.); nataqain@ukm.edu.my (S.N.B.); 2 School of Environmental and Natural Resource Sciences, Universiti Kebangsaan Malaysia, 43600 Bangi Selangor, Malaysia; ntalip@ukm.edu.my

**Keywords:** Taro viruses, Dasheen mosaic virus, Taro bacilliform virus, Colocasia bobone disease virus, Taro vein chlorosis virus

## Abstract

Taro is an important crop in parts of the world, especially in the Pacific Islands. Like all plants, it is also susceptible to virus infections that could result in diseases, which negatively affects the source of food and trade revenue. Understanding the biology of taro viruses could improve current knowledge regarding the relationship between viruses and taro, thus allowing for a better approach towards the management of the diseases that are associated with them. By compiling and discussing the research on taro and its four major viruses (*Dasheen mosaic virus*, *Taro bacilliform virus*, *Colocasia bobone disease virus,* and *Taro vein chlorosis virus*) and a relatively new one (*Taro bacilliform CH virus)*, this paper explores the details of each virus by examining their characteristics and highlighting information that could be used to mitigate taro infections and disease management.

## 1. Introduction

Taro (*Colocasia esculenta* (L.) Schott) is a perennial root crop from the family *Araceae* (common name: Aroids) with substantial socio-economic importance in tropical and sub-tropical regions, especially Southeast Asia and the Pacific Island countries [1,2,3]. Aside from its edible corms, this crop has significant cultural value for its communities [4,5,6,7]. Taro has been found globally in various regions and countries, including but not limited to, Hawaii, the Caribbean, American Samoa, Papua New Guinea, Southern Africa, Malaysia, Bangladesh, and Australia, with local common names, such as dasheen, kalo, talo, and keladi [3,8,9,10,11,12,13,14,15]. The plant has also been described as invasive species in some parts of these regions [3]. Local cultivation—almost exclusively through vegetative propagation—is often preferred, since it is much less expensive than imports [6]. As such, taro cultivation is an important aspect of the economy, although many viruses and diseases affect the crop [16]. Taro viruses, which could lead to diseases, damage the crop yield and sometimes lead to plant death [17]. Multiple viruses have been found to infect taro, including the four main viruses; *Dasheen mosaic virus* (DsMV)*, Taro bacilliform virus* (TaBV)*, Colocasia bobone disease virus* (CBDV)*,* and *Taro vein chlorosis virus* (TaVCV) [5,6,8,9,17,18,19,20,21,22,23,24,25,26] that this review will centre on. This review will also mention a relatively new virus *Taro bacilliform CH virus* (TaBCHV) [17,19,27].

Meanwhile, the widely studied taro diseases are Alomae and Bobone, which are found to be restricted to the Solomon Islands and Papua New Guinea [5,28]. Previous studies implied that the co-infection of two different viruses could result in symptomatic plants; co-infection of TaBV and CBDV result in the Alomae, while infection by CBDV alone results in the Bobone [5,29]. In addition, plants with Alomae symptoms are also found to contain another putative rhabdovirus, TaVCV, although confirmation tests for correlation are yet to be done [22]. 

Specific studies have focused on reporting, understanding, and analysing these individual viruses and the respective effects on taro in a particular region, which pave the way for nucleotide sequencing of the viruses. Meanwhile, there are broader studies that focused on multiple taro viruses, which highlighted the comparison between the viruses and speculated on how they interact with each other in infected individuals. The general gap between the specific and broader studies is that there is a need of a more in-depth overview of all the viruses, alongside a future direction for taro virus studies; be it for further sequencing or disease mitigation. This review aims to fill this gap by exploring the characteristics of the viruses through an examination of the morphology, origin, symptoms, genome integration, and to provide insight into taro management strategies.

## 2. Dasheen Mosaic Virus 

*Dasheen mosaic virus* (also known as *Dasheen mosaic potyvirus*) is a positive-sense, single-stranded RNA virus that belongs to the family *Potyviridae* with typical host range being the members of *Araceae* including taro, cocoyam, and caladium. A particular characteristic of DsMV is that the virus has a considerably large capsid protein (CP) compared to other potyviruses. Although there are serological similarities between different DsMV isolates, they have different CP sizes and the symptom severity—foliar symptoms, chlorotic spots, more pronounced stunting—on host species varies. However, any direct correlation between CP sizes and the severity of different isolates has not been shown [30]. Furthermore, different CP sizes do not appear to interfere with the diagnosis of DsMV [30,31,32].

**Figure 1:**
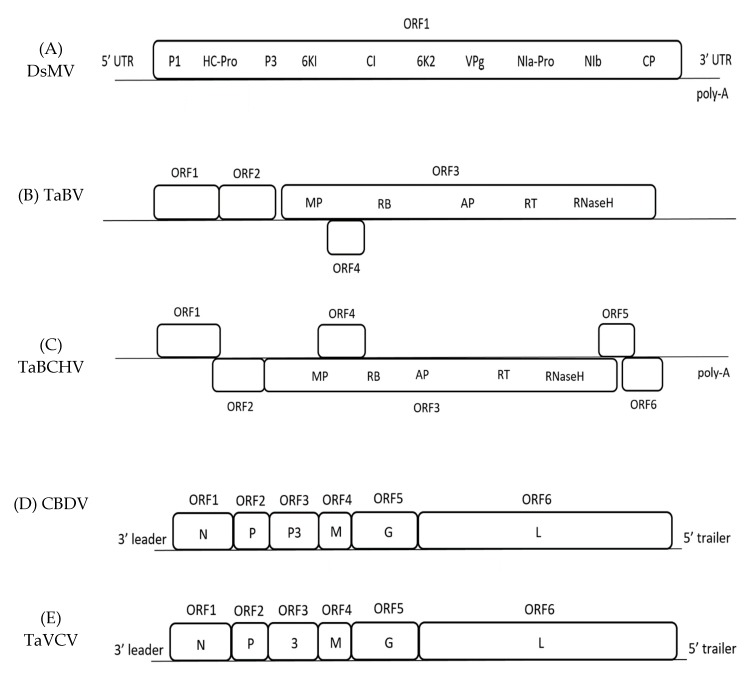
Schematic representation of the genome organization of each viruses. (**A**) Genome organisation of DsMV showing one open reading frame (ORF) encoding 10 putative proteins. (**B**) Genome organisation of TaBV showing four ORFs, with ORF3 encoding for domains homolog to Movement Protein, zinc-finger like RNA binding domain, Aspartic Protease, Reverse Transcriptase, and Ribonuclease H. (**C**) Genome organisation of TaBCHV which is similar to TaBV, with extra ORFs 5 and 6. (**D**) Genome organisation of CBDV encoding 6 ORFs with the respective domain; N, nucleocapsid gene; P, phosphoprotein gene; P3, putative movement protein gene; M, matrix protein gene; G, glycoprotein gene; L, polymerase gene. (**E**) Genome organisation of TaVCV, similar to CBDV, but ORF3 encoding for gene 3 instead.

The genome of DsMV is made up of one open reading frame (ORF) that encodes a polyprotein, alongside a 5’- untranslated region (UTR) and a 3’- UTR ending with a poly-A tail. The polyprotein is further cleaved into 10 proteins; P1, HC-Pro, P3, 6K1, CI, 6K2, VPg, NIa-Pro, NIb, and CP (Figure 1) [33,34]. So far, six complete nucleotide sequences of DsMV isolates have been sequenced and recorded in National Centre for Biotechnology Information (NCBI) online database, with the first DsMV sequence ever being recorded from China (Table 1) [35]. 

**Figure 2:**
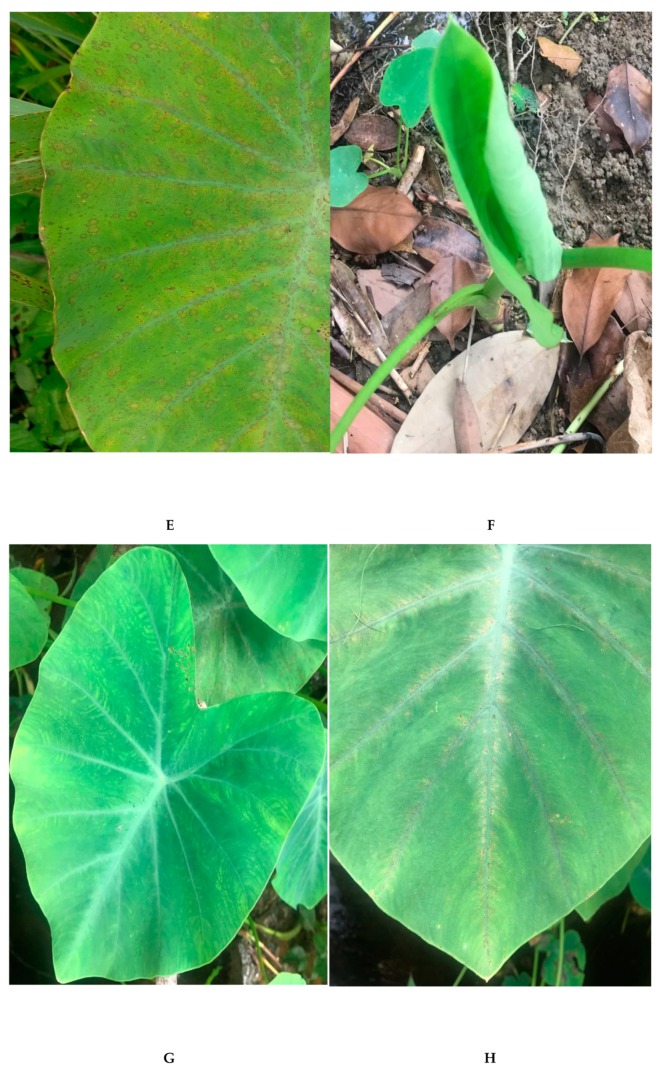

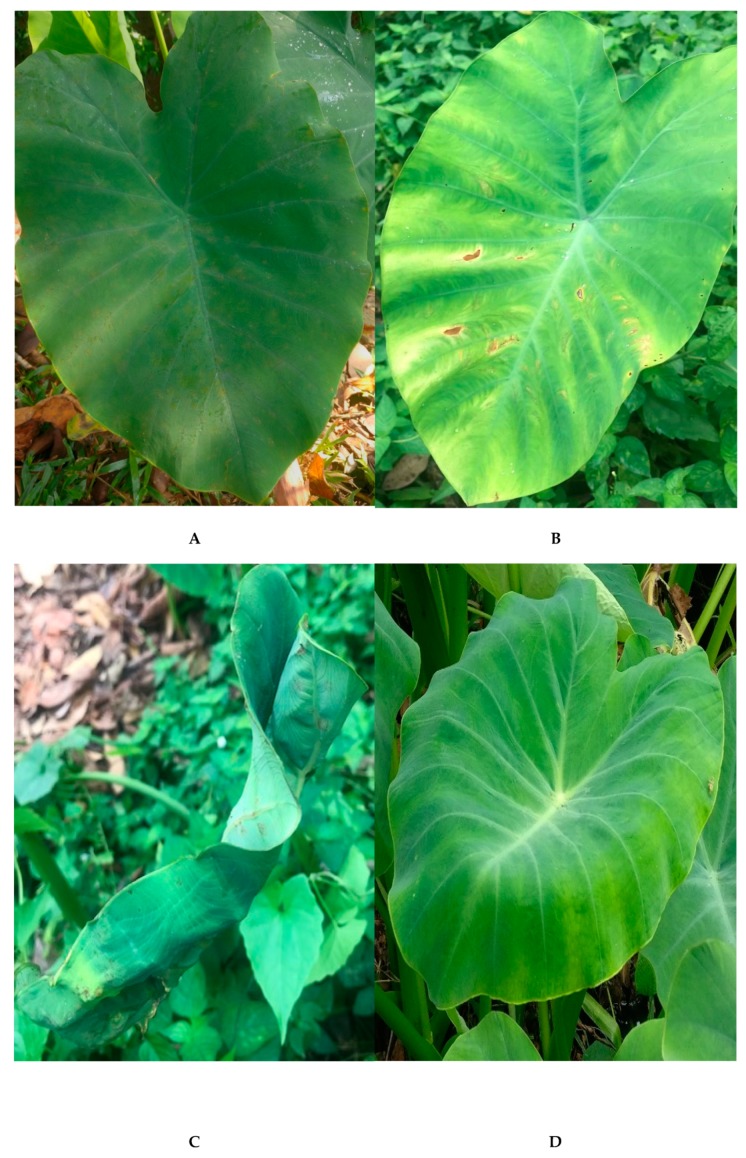
Taro leaves. (**A**) Relatively healthy taro leaf; DsMV infection symptoms: (**B**) conspicuous colouration, (**C**) leaf malformation; TaBV symptoms: (**D**) leaf curling downwards; TaBCHV symptoms: (**E**) foliar brown spots; CBDV symptoms: (**F**) stunting, (**G**) thickened vein and leaf blades; TaVCV symptom: (**H**) vein chlorosis.

Taro plants that were infected with DsMV have the typical feather mottle symptoms with conspicuous mosaic patterns—yellow, whitish patterns against the green; pale green against dark green—and sometimes malformation and dwarfing of the leaves (Figure 2) [6,31]. Since the majority of cultivated taro are vegetatively propagated, they indefinitely carry the virus once they have been infected [20,30]. DsMV has been presumed to be present wherever cultivation of taro occurs, as it has been found in many countries where the crop is grown [6,30,33,36,37,38]. Multiple publications on DsMV-taro have not only allowed for genome characterisations, diagnostics assays, and symptomatology to be investigated, but has also led to the potential development of DsMV-free taro cultivars through thermotherapy and tissue culture [20,31]. The interaction between the virus and other hosts is also a research interest, as there is speculation regarding the extent of DsMV that is present in taro as a result of cross-species transmission, via mechanical transmission or aphid vector that feeds on different host plants [6,39]. This adds another level of complexity in mitigating the management strategies of DsMV and taro diseases. 

## 3. Taro Bacilliform Virus

*Taro bacilliform virus* is an aroid-specific pararetrovirus from the family *Caulimoviridae* and it has been classified in the genus *Badnavirus*. This classification is based on the examination of bacilliform-shaped virions that were found in the infected plants and the mealybug transmission studies [5,40,41,42,43]. Other examples of badnaviruses include *Banana streak virus* (BSV), *Dioscorea bacilliform virus* (DBV), and *Sugarcane bacilliform virus* (SCBV), each with their own host range. This wide host range of badnaviruses has been the subject of study due to the suitability of the promoter for transgenic experiments [44]. 

The first complete nucleotide sequence of TaBV was recorded in Papua New Guinea (PNG), with four isolates that were found later in Australia and East Africa (Table 1) [5,27,45]. The PNG isolate has been found to contain 7458 bp consisting of four ORFs (Figure 1); ORF 1–3 are comparable to other badnaviruses, while ORF 4 is only comparable to ORFs of the atypical badnaviruses *Citrus yellow mosaic virus* (CYMV) and *Cacao swollen shoot virus* (CSSV) [5]. The function of the ORF 1 protein is unknown, whereas ORF 2 contains sequences that may be involved in virion assembly [5]. The ORF 3 of TaBV is a putative sequence that includes motifs, such as the movement protein (MP), CP, aspartic protease, reverse transcriptase (RT), and ribonuclease H (RNase H) [5,45]. ORF 4 contains a sequence of a putative protein with minimal homology to any published sequences [45]. Additionally, the sequence of TaBV includes the highly conserved tRNA^met^-binding site and TATA sequence [5,27]. 

The symptoms of TaBV in taro include vein chlorosis, stunting, and leaves curling downwards (Figure 2) [4]. The virus is transmissible through three methods—the mealybug vector, vegetative propagation, and via seeds or pollen—while no mechanical transmission has so far been reported [41,45,46]. However, a study has shown that TaBV-infected plants may or may not exhibit symptoms, where the majority of the symptoms are observed on the youngest leaves [46]. The existence of symptomless plants with TaBV leads to speculation that the virus must be present with another virus to allow for the plants to be symptomatic [4]. This is the case for TaBV being present alongside a putative rhabdovirus, CBDV, which leads to the lethal Alomae disease [5,47]. However, another serological study found that bacilliform-like particles only exist in the insect vector, but not in the infected plants [46]. The virus latency would prove to be a challenge in diagnosis, as a small amount of virus could still remain in a community without exhibiting any symptoms, and, when combined with vegetative propagation, the virus could rapidly spread into the crop community [29].

Besides TaBV, there is another badnavirus infecting taro, *Taro bacilliform CH Virus* (TaBCHV). The first complete nucleotide sequence was recorded in China, before others in the United States in America (USA) and East Africa (Table 1) [17,19,27]. TaBCHV contains six ORFs, with ORF1-4 being comparable to the ORFs of TaBV (Figure 1) [19]. TaBV and TaBCHV show <80% similarity in genome structure and RT/RNase sequence [19]. In addition, TaBCHV has also been detected to contain putative tRNA^met^-binding region and a potential TATA sequence. In contrast to TaBV, TaBCHV also contains ORF5 and ORF6—both potentially encoding proteins that are yet to be elucidated—and a poly-A tail [17,19]. The symptoms of the virus include feathery mosaic symptom and foliar brown spots (Figure 2) [9,19]. So far, no vector transmission studies have been done on the virus. Overall, comparative studies could be done on TaBV and TaBCHV.

## 4. Colocasia Bobone Disease Virus

*Colocasia bobone disease virus* is a rhabdovirus classified in the genus *Cytorhabdovirus,* where replication occurs in the cytoplasm. The rhabdovirus can be found in typical taro that is infected with bobone disease, being characterised by the occurrence of bacilliform-shaped virions in sap dips [40]. The first and only completed nucleotide sequence of such virus so far was found in the infected taro of the Solomon Islands (Table 1). Although the paper on the complete genome sequence initially stated that it was a CBD-associated virus rather than the actual CBDV, the characteristics are highly suggestive that it is indeed CBDV, although further confirmation studies should be done [47]. CBDV is a negative-strand RNA genome that consists of a complementary 5’- and 3’- UTR bordering six major ORFs encoding the sequence of the nucleocapsid gene (N), phosphoprotein gene (P), putative movement protein gene (P3), matrix protein gene (M), glycoprotein gene (G), and polymerase gene (L) (Figure 1) [47].

The virus has been related to the infamous taro diseases, Alomae and Bobone, with several studies confirming the presence of CBDV in taro infected by either of the diseases, although the direct causality remains unclear [22,46]. Both of the diseases cause the plants to exhibit symptoms, such as stunting, gall formation in petioles, plus thickened veins and leaf blades (Figure 2). The symptoms that were observed in plants with Alomae also include chlorosis, unfurled leaves, and systemic necrosis, ultimately leading to plant death. Meanwhile, plants with Bobone could remain green and eventually recover [22,40]. The transmission of the virus is known to be via the planthopper *Tarophagus proserpina,* which acts as a vector for bacilliform particles alongside mealybugs [41,47].

The range of CBDV distribution remains unknown, with the only identification through electron microscopy so far being in the taro of Solomon Islands and PNG. The correlation between this locality with the restriction of Alomae and Bobone in these regions remains to be proven, although it seems likely [40,47]. 

## 5. Taro Vein Chlorosis Virus

*Taro vein chlorosis virus* is a rhabdovirus that is characterised by the presence of bacilliform-like virions in sap dips, similar to CBDV [22]. TaVCV and CBDV are both rhabdoviruses that encode five similar major proteins; N, P, M, G, and L (CBDV contains P3 instead of the 3 in TaVCV) (Figure 1). TaVCV belongs in the genus *Nucleorhabdovirus*, where replication occurs in the cell nucleus. Additionally, they are both serologically different from each other [48]. The only nucleotide sequence of TaVCV completed so far is the one identified in Fiji (Table 1). 

The most apparent symptom of plants that were infected with TaVCV is leaf-vein chlorosis, especially at the leaf margin (Figure 2). This habitually leads to necrosis [8,22]. By examining the records for symptomatic plants and electron microscopy, the distribution of the virus covers the Pacific Islands region, although the virus is not mechanically transmissible and any involvement of vector remains elusive [8,48,49]. The wide distribution of the virus is posited to be by the spread of the infected plant materials, which is a common practice in taro cultivation [8].

## 6. Taro as Host for Plant Viruses

### 6.1. Diagnostics

Various detection tests can be used to detect all of the viruses in this review, with the polymerase chain reaction (PCR)-based assay being the most used in studies, which is potentially due to it being more efficient [30,53,54]. It is found that the PCR-based assay is proficient, even for virus isolates with high serological and genomic variability [4,5,54]. Besides that, other methods, including western blot, immuno-osmophoresis, sodium dodecyl sulfate-polyacrylamide gel electrophoresis (SDS-PAGE), enzyme-linked immunosorbent assay (ELISA), and dot-blot hybridisation assay can also be used (Table 2) [9,22,30,31,32,47,54].

Detection assays are essential in the screening of taro germplasms, ensuring that they are virus free and suitable for movement across the border [4,5,54]. 

### 6.2. Taro-Virus Interaction

A successful viral infection usually occurs due to the viral genome containing ORFs that could encode the proteins that are necessary for host infections [56]. The genome structure of the viruses in this review encodes putative proteins that are essential in the establishment of a positive infection [5,19,33,47,48]. The majority of the putative proteins are typical across virus families, such as the MP and RT of the TaBV and TaBCHV, where both of the proteins play a major role in transport mechanism and replication of the viral genome inside the host, respectively [5,19,56,57]. Meanwhile, the genome of CBDV and TaVCV are both found to encode for L polymerase, which is highly likely to be responsible for the enzyme catalysing RNA replication in taro [47,48]. DsMV genome encodes HcPro, among others, which has been described as a regulator for potyvirus pathogenicity, implying its role in the host-virus arms race [33,58].

Proteins that are encoded by the viral genome could be directly responsible for host infections. As such, understanding the functions of ORFs and the proteins could illuminate the molecular interaction of taro and the viruses. However, potentially due to its complexity, studies regarding this interaction remains elusive. 

### 6.3. Taro-Virus Genome Integration

Previous work speculates that the genome of TaBV could integrate into the taro genome [4,46,59]. An electron microscopy study by Yang and colleagues found ubiquitous TaBV sequence in infected taro, further suggesting that integrants could be formed [4]. This is similar for other badnaviruses, such as the BSV infection in banana cultivars, where interspersion of the viral genome in the host chromosomal DNA was evident, suggesting that genome integration between the virus-host does indeed occur [4,60]. Genome integration could add another layer of complexity to the diagnosis of viruses in taro germplasm, as the contemporary methods might not be suitable in diagnosing both the autonomous viral sequence and the integrated ones [4,19,59].

### 6.4. Taro Diseases

As mentioned before, Alomae and Bobone are the major diseases infecting taro [5,28]. It has been found that the virus particles that are responsible for Alomae are only present in the male taro cultivars, whereas the ones responsible for Bobone only occur in female cultivars [28,29,61]. Additionally, the particle size in Alomae is smaller than the ones in Bobone, although the cultivars infected with Alomae often leads to death while Bobone-infected cultivars can recover [29,62]. 

Due to its nature, Alomae is considered to be the most lethal taro disease that significantly affects the crop [16,29]. A study suggests that the taro cultivars of various origin possess little to no resistance to Alomae, as most of the infections lead to plant death [41]. As such, it is vital that the geographical movement of taro planting materials from the Solomon Islands and Papua New Guinea is controlled [4,23,29,41].

### 6.5. Taro Viral Disease Management and Challenges

If left uncontrolled, the taro diseases could potentially be widely distributed and the outcomes would be devastating to the taro crop industry. Hence, a certain degree of disease management and control is essential in maintaining a considerable amount of taro safe for consumption. One of the most important approaches is to ensure the better supervision of the movement of germplasms by authorities (and researchers) across the border, especially with regards to symptoms identification and diagnostic assays [54]. As such, adhering to the international guideline for taro plant movement is important, with the suggested plant material usually being the virus-screened plant tissue culture [63]. Additionally, the development of virus-free taro is vital in maintaining the supply of healthy taro for crop growers, and although countless studies have been done—mainly through transgenic and genetic engineering approaches—progress remains unhurried [2,19,31,46,64,65,66,67].

A relatively common method of management is roguing, which is the periodic physical removal of infected plant parts. It is an established viral disease control strategy and used in various plant species to protect against respective viruses, although it can be quite tedious [29,68]. At times, the removal of the entire infected individuals is necessary to best control the viral transmission. However, crop growers might be hesitant to do so, as the crops are valuable and it might go against the traditional practices of growing taro [29,68]. The local practices by the crop growers also do not accommodate the use of insecticides, which could directly control the vector population [29,68]. The application of insecticides, if possible, needs to be frequently done at a different taro growth stage and this might bring undesired effects to the biodiversity [29]. 

Apart from that, there is an approach to introduce the intercropping of non-host plants among taro cultivars [11]. This method could potentially curb the distribution of the virus. However, understanding the entire biology of the species and virus involved is required, and that would take a long time before any positive results could be observed [11,68]. In addition, intercropping has always been a part of the integrated management strategies where all the relevant approaches are used for cultivation of crops [11,68]. 

Another approach is to use biological control of the vector population. Using predators to control the vectors spreading viruses on taro showed potential, considering the population of said vectors is still relatively small [16]. Few studies imply that a bug, *Cyrtorhinus fulvus,* could effectively control the *Tarophagus* population, although no research has been done to further explore this method in the last decade [16,69].

Observation from another study found the number of plants with Alomae symptoms was reduced in the colder months when compared to warmer ones, highlighting a possible factor of the timing and temperature of the soil and surroundings, although this observation could be simply due to the virus inactivity rather than its complete removal [29]. However, this is perhaps not applicable in the tropics and sub-tropics, where major cultivation of taro occurs [29].

## 7. Conclusions

This review has outlined the characteristics of viruses infecting taro plants. It is essential for the taro planting materials to be virus-free, which is best achieved through the development of suitable diagnostic assays and the careful screening of such materials across the border. Although traditional methods of cultivation may prove to be a challenge in the management of taro viruses, continuous exposure to current information regarding taro could help local farmers, especially the next generation of modern farmers, to better fortify their crops against diseases. The bridge that was formed between current research, farmers, and local authorities could improve overall the understanding of taro viruses and diseases that are associated with them, thus providing an insight regarding the best way to mediate the problem. This would not only be beneficial to the local consumers, but could help to sustain the crop-based economy of taro growing regions.

## Figures and Tables

**Table 1:** The isolates with complete genome sequence for the four main viruses (*Dasheen mosaic virus* (DsMV), *Taro bacilliform virus* (TaBV), *Colocasia bobone disease virus* (CBDV), *Taro vein chlorosis virus* (TaVCV)) and *Taro bacilliform CH virus* (TaBCHV) infecting taro (*C. esculenta*).

Name (Taxon)	Isolate (Length)	Source	Accession Number	Reference
DsMV (Potyvirus) (+)ssRNA virus	DsMV (10038 bp)	China	NC_003537.1	[35]
	DsMV strain I (10002 bp)	Hawaii, USA	KY242358.1	[33]
	DsMV strain II (10019 bp)	Hawaii, USA	KY242359.1	[33]
	DsMV CTCRI-II-14 (10004 bp)	India	KT026108.1	[50]
	DsMV SdP (10030 bp)	China	JX083210.1	[51]
	DsMV T10 (10024 bp)	India	KJ786965.1	[52]
TaBV (Badnavirus) ssDNA virus	TaBV (7458 bp)	Papua New Guinea	NC_004450.1 AF357836.1	[5]
	TaBV Aus7 (7494 bp)	Australia	MG017318.1	[45]
	TaBV Ke52 (7805 bp)	East Africa (Kenya)	MG017321.1	[27]
	TaBV Tz17 (7803 bp)	East Africa (Tanzania)	MG017322.1	[27]
	TaBV Tz24 (7798 bp)	East Africa (Tanzania)	MG833013.1	[27]
	TaBV Ug75 (7796 bp)	East Africa (Uganda)	MG017323.1	[27]
TaBCHV (Badnavirus) ssDNA virus	TaBCHV-1 (7641 bp)	China	NC_026819.1	[19]
	TaBCHV-2 (7641 bp)	China	KP710177.1	[19]
	TaBCHV Et17 (7610 bp)	East Africa (Ethiopia)	MG017324.1	[27]
	TaBCHV Ke43 (7647 bp)	East Africa (Kenya)	MG017325.1	[27]
	TaBCHV Tz27 (7389 bp)	East Africa (Tanzania)	MG833014.1	[27]
	TaBCHV Tz36 (7654 bp)	East Africa (Tanzania)	MG017326.1	[27]
	TaBCHV Ug10 (7643 bp)	East Africa (Uganda)	MG017327.1	[17]
	TaBCHV isolate Hawaii (7634 bp)	Hawaii, USA	KY359389.1	[17]
CBDV (Cytorhabdovirus) (-)ssRNA virus	CBDV strain SI (12193 bp)	Solomon Islands	NC_034551.1 KT381973.1	[47]
TaVCV (Nucleorhabdovirus) (-)ssRNA virus	TaVCV (12020 bp)	Fiji	NC_006942.1 AY674964.1	[48]

**Table 2:** Summary of the detection methods for taro viruses.

Virus	Detection Method	Remarks	Reference
DsMV	SDS-PAGE	Estimation on the relative size of CP of DsMV compared to other viruses could be done.	[32]
	Western blot	Characterising the CP of DsMV could be done for comparative studies among isolates and potyviruses.	[30]
	ELISA	Relatively sensitive & easy to use for routine virus detection.	[37]
	RT-PCR	This method was developed after nucleic acid sequence was completed. This allow the detection of wide range of isolates.	[31,33,35,40,54]
TaBV	PCR	Primers designed are usually based on the putative coding regions of TaBV, with the most widely mentioned in literatures being the primers BadnaFP & BadnaRP responsible for RT & RNaseH-coding regions.	[4,5,27,45]
TaBCHV	RT-PCR	Developed to examine the occurrence & distribution of TaBCHV on taro.	[9,17,19]
	Dot-blot hybridisation assay	Used in confirmation test alongside RT-PCR in [9].	[9]
	PCR	In [27], the method is described alongside rolling circle amplification (RCA) to identify the virus.	[27]
CBDV	Immuno-osphoresis	A part of serological study of rhabdoviruses detection in multiple taro samples in the Pacific Islands.	[22]
	RT-PCR	Method used for detection, completing genome sequence, and recording distribution of the viruses.	[40,47]
TaVCV	ELISA	A part of serological study of rhabdoviruses detection in multiple taro samples in the Pacific Islands.	[22]
	RT-PCR	For examining the distribution of viruses in the Pacific Islands.	[54,55]
